# Prior Restorative Procedures to Endodontic Treatment

**DOI:** 10.7759/cureus.37106

**Published:** 2023-04-04

**Authors:** Mário A Moreira, Virgínia R Silveira, Veronica O Alcantara, Frederico B Sousa, Bruno C Sousa

**Affiliations:** 1 Dentistry, Ceará Federal University, Sobral, BRA; 2 Morphology, Paraíba Federal University, João Pessoa, BRA

**Keywords:** clinical success, dental fracture, endodontics, periodontics, composite resin restorations, chatgpt

## Abstract

Tooth loss due to fracture and the failure of endodontic treatment (ET) are common situations in teeth with extensive tissue destruction. This is due to the fragility of the remaining dental structure and the difficulty in sealing cavities, which is sometimes associated with the violation of the supracrestal insertion tissue. The previous restoration of marginal ridges or cusps with composite resin (CR) restores their fracture resistance, due to the adhesive characteristics of the restorative material, while also protecting the quality of endodontic treatment through better sealing. However, the protocol adopted in teeth requiring endodontic treatment involves performing the restorative procedure only after the endodontic procedures. The objective of this study was to report a case in which restoration of marginal ridges and/or cusps was performed prior to endodontic treatment, focusing on maintaining the tooth in function without dental fracture. The restoration was performed with an inverted operative sequence before the endodontic treatment. There was a violation of the supracrestal insertion tissue, requiring crown lengthening surgery (CLS) prior to the restorative procedure. Clinical and radiographic evaluations were performed postoperatively at seven days, three, six, and nine months, and five years. Tooth function was maintained without dental fractures or restoration loss. Periradicular space healing occurred with the disappearance of the lesion. Performing the restorative procedure prior to endodontic treatment in teeth with extensive coronal destruction is an alternative technique that facilitates clinical procedures, reduces the likelihood of dental loss due to fracture, and promotes endodontic treatment success.

## Introduction

The reduction of dental structure caused by caries, fractures, and access cavity preparations, and the consequent decrease in fracture resistance, have a negative influence on remaining teeth, highlighting the importance of the amount of remaining walls at the time of endodontic access [1].

Endodontically treated teeth have significantly different biomechanical properties compared to vital teeth [2]. Configuration, isthmus width and cavity depth are highly critical factors in determining the risk of fracture [3]. Fractures in endodontically treated teeth are quite common clinical situations and may have several etiological factors [4]. Loss of coronary structure caused by caries cavities, in addition to the access cavity prior to endodontic therapy, are relevant predisposing factors [5]. Enlargement of the coronal third of the root canal space is considered an important procedure in endodontic therapy and in the use of fiber posts [4]. However, extensive dentin removal weakens the root structure and may reduce the fracture resistance of teeth [4]. Special attention to preserve sufficient remaining dentin should be given to the maxillary premolars most susceptible to fracture [6]. The remaining coronal tooth structure is a decisive factor for the optimal biomechanical behavior of endodontically treated teeth [7]. The reliable adhesive dental techniques available have expanded the restorative options for endodontically treated teeth [8].

Pre-endodontic restorations refer to dental restorations that are placed before root canal therapy is performed. These restorations are essential to maintaining the structural integrity of the tooth and reducing the risk of tooth fractures [9].

When a tooth is affected by caries or trauma, it can lead to a weakening of the tooth structure. Pre-endodontic restorations, such as fillings or dental crowns, are used to restore the damaged structure of the tooth and provide additional support to the tooth. This helps prevent further damage and maintains the natural tooth for a longer period [9].

The conventional operative sequence, in which the restorative treatment (RT) occurs after the endodontic treatment (ET), favors the persistence of tooth fragility for a longer period (due to the lack of reinforcing structures such as marginal ridges and/or cusps), which may increase the prevalence of dental fractures before, during, and after endodontic treatment [10]. In the presence of large coronal destructions, other disadvantages may be cited, such as difficulty in achieving absolute isolation, which can cause trauma to the gingival tissues, the undesirable presence of oral fluids in the operative field, and interference in the adhesion between the composite resin (CR) and the dentin due to collagen fiber modification by the action of hypochlorite [11].

Restorations of endodontically treated teeth (RETT) can recover up to 72% of the fracture resistance of teeth compared to those with intact dental crowns [10]. Thus, performing these restorations prior to ET would contribute to the increase in tooth resistance, reducing the risk of fracture during the operative sequence, from waiting for ET to performing the definitive RT, since there is a positive correlation between the remaining dental surface area and the fracture resistance [1].

An important factor to consider before performing pre-endodontic restorations is the evaluation of the periodontal condition. The RT should always aim for the health of the periodontal tissues and respect the re-establishment of the biological space, designated by the latest classification (2018) of the American Academy of Periodontology (AAP) as the supracrestal insertion tissue [12]. Subgingival restoration margins invading the junctional epithelium space and the supracrestal connective tissue attachment may be related to inflammation and clinical attachment loss, associated or not with gingival margin recession [13]. Thus, one of the ways to recover biological width before RT may be through surgical crown lengthening.

The aim of this study was to report a case in which restoration was performed by recovering the supracrestal space prior to endodontic treatment, observing the maintenance of the treated tooth function, the longevity of the restorative procedure, the absence of dental fracture, and periradicular region healing.

## Case presentation

The study was approved by the Research Ethics Committee of the Vale do Acaraú State University (protocol #501.950/2013). The patient signed the informed consent form and was selected from the spontaneous demand of the Dental Clinic of the Federal University of Ceará (UFC), Sobral Campus.

**Figure 1:**
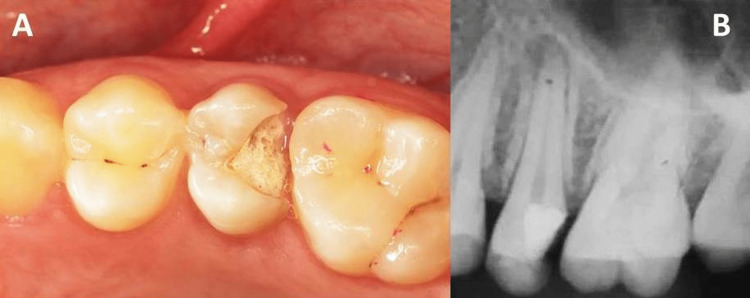
(A) Extensive caries lesion in the upper left second premolar and (B) radiograph of the upper left second premolar showing involvement of the pulp chamber and periapical lesion.

Patient with toxic shock syndrome (TSS), 27 years old, attended the institution's clinic for dental treatment. There was no complaint of pain or any type of dysfunction. Clinical examination showed the presence of an extensive caries lesion in the upper left second premolar. Extensive loss of coronal structure involving the distal marginal ridge and almost the entire occlusal surface, compromising about a third of the crown reinforcement structure. The tooth was diagnosed with chronic apical periodontitis, and endodontic treatment was necessary (Figure 1). After careful analysis, it was decided that direct restorative treatment was viable, as there were two walls present for adhesion with dental enamel. The patient had a good periodontal condition, excluding the dental element in question, where there was a violation of the supracrestal insertion tissue space and an indication of crown lengthening surgery (CLS).

The procedures were performed in a clinical sequence distributed over three sessions, in order to restore the reinforcing walls through the restoration with CR, leaving only the occlusal access for subsequent endodontic treatment, and definitive coronal sealing (Table 1).

**Table 1:** Procedures performed and sequences in the suggested protocol for treatment with pre-endodontic restorations.

First session	Second session	Third session
Clinical examination, periapical, and interproximal radiographs; color selection for composite resin restoration; periodontal surgery for crown lengthening; composite resin restoration; endodontic access; suturing	Suture removal, postoperative periodontal clinical evaluation, evaluation of composite resin restoration integrity	Endodontic treatment, final composite resin restoration (occlusal view), final radiograph

**Figure 2:**
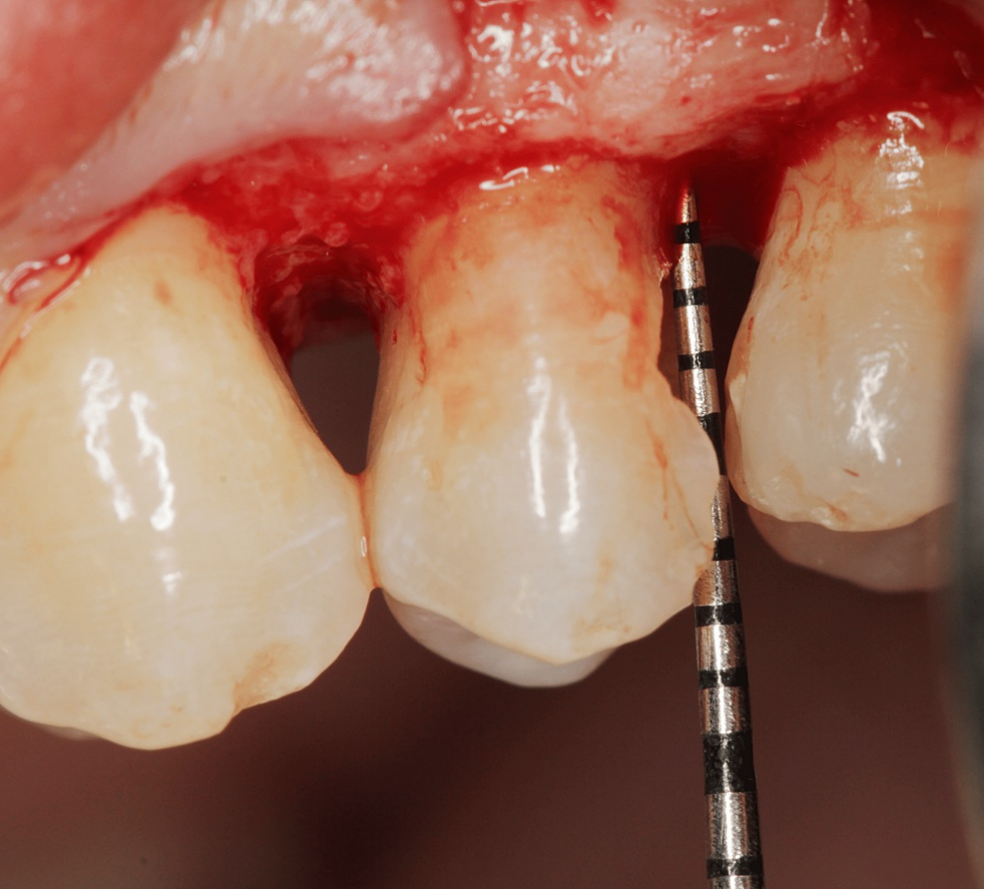
Osteotomy was performed until a distance of 3 mm between the end of the preparation and the marginal bone crest was re-established.

In the first session, the color selection was made using the color scale (VITA, Bad Säckingen, Germany), and infiltrative anesthesia with 2% mepivacaine (DFL, Rio de Janeiro, Brazil) was used. For the CLS periodontal surgical procedure, a modified Widman flap was performed through an intra-sulcular incision using a no. 3 scalpel handle and a 15c blade (Lamedid, Barueri, São Paulo). The flap was fully retracted with Free and Molt elevators (Trinity, São Paulo, Brazil), followed by the removal of granulation tissue with a Crane-Kaplan Curette (Trinity, São Paulo, Brazil). The amount of bone to be removed for the re-establishment of the supra-crestal insertion tissue was measured with a PCP-UNC 15-millimeter probe (Millennium Golgran, São Caetano do Sul, Brazil). Osteotomy was performed with Fedi and Rhodes chisels and Buck and Schluger files (Trinity, São Paulo, Brazil) until a distance of 3 mm between the end of the preparation and the marginal bone crest was re-established (Figure 2).

After osteotomy, absolute isolation of the operative field was performed, followed by direct restoration of the dental element, reconstructing the marginal crests and cusps. The following materials were used: 37% phosphoric acid (3M ESPE, São Paulo, Brazil)-3 ml, Adper Single Bond 2 adhesive (3M ESPE, São Paulo, Brazil)-5.6 ml, and Filtek Z350 XT composite resin (3M-ESPE, São Paulo, Brazil)-4 g. The CR was performed using the matrix and matrix holder set or unimatrix clamp (TDV, São Paulo, Brazil) and a sectioned matrix (TDV, São Paulo, Brazil) with the aid of wooden wedges to re-establish contact points. A gutta-percha cone (Dentsply Sirona, São Paulo, Brazil) with vaseline was used in the root canals to prevent the obliteration of these canals with composite resin.

**Figure 3:**
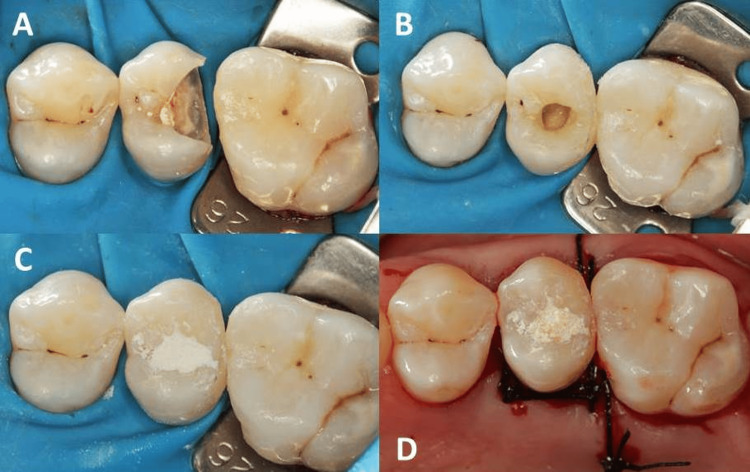
Operative sequence, first session: (A) absolute isolation of the operative field, (B) pre-endodontic restoration of the dental element, reconstructing the marginal crests and cusps, (C) provisional coronal sealing after endodontic access, (D) after removal of the rubber dam, the detached flap was stabilized through vertical interrupted mattress suture and simple suture.

After the pre-endodontic restoration was completed and with the endodontic access properly preserved, root canal catheterization was performed using K-File files (Dentsply Sirona, São Paulo, Brazil), irrigation (2.5% sodium hypochlorite) (Asfer, São Caetano do Sul, Brazil), intracanal medication with Tricresol Formalin in a cotton pellet (Biodinâmica, Ibiporã, Brazil), and provisional coronal sealing (Figure 3) with Coltosol® (Coltene, Rio de Janeiro, Brazil).

After removal of the rubber dam, the detached flap was stabilized through vertical interrupted mattress suture and simple suture with 5.0 Best Care nylon thread (Biodinâmica, Ibiporã, Brazil). The occlusion of the restoration was tested, and when necessary, an occlusal adjustment was performed. Postoperative care was provided through the prescription of systemic analgesic medication Sonridor CAF (GlaxoSmithKline, Rio de Janeiro, Brazil) and chemical control with 0.12% chlorhexidine digluconate (Colgate, São Paulo, Brazil).

The second clinical session was performed two weeks after the procedures. At that time, the suture was removed, and the healing condition was properly observed. The pre-endodontic restoration was polished using diamond tips 1190F, 1190FF, 3195F, 3195FF, 3118F, 3118FF, 3168F, 3168FF, multilaminated trunk-conical drill kit (KG Sorensen, Cotia, SP, Brazil), siliconized flame-shaped tips, felt wheels, sandpaper strips, and diamond pastes (TDV, Pomerode, Santa Catarina, Brazil).

The third clinical session occurred one week later. Coronal access was performed, rubber dam isolation, removal of the cotton pellet, canal irrigation with 2.5% sodium hypochlorite (Asfer, São Caetano do Sul, Brazil), pre-enlargement with K-file files #15, #20, and #25 (Dentsply, São Paulo, Brazil), and Gates Glidden 3 and 2 (Dentsply, São Paulo, Brazil). Next, odontometry was performed with I-Root® electronic apex locator (Meta-Biomed, Tokyo, Japan), canal instrumentation with Flexofile first series files (Dentsply, São Paulo, Brazil) allowing apical preparation, and K-file second series files (Dentsply, São Paulo, Brazil) allowing the execution of the stepped back preparation. Root canal filling was performed with gutta-percha cones (Dentsply, São Paulo, Brazil) and MTA Fillapex endodontic cement (Angelus, Londrina, Brazil).

**Figure 4:**
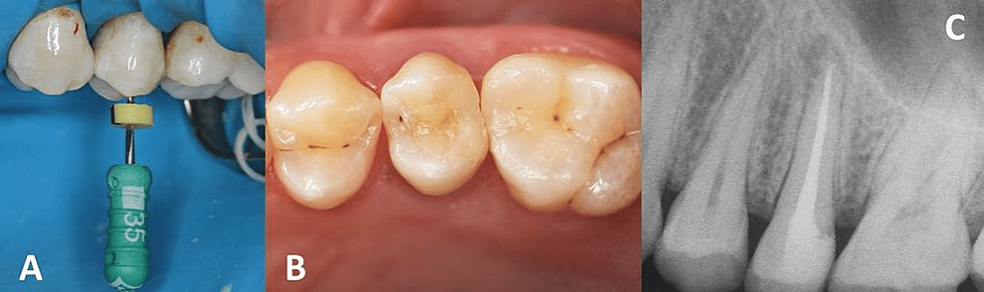
(A) Preparation of the root canals, (B) definitive post-endodontic restoration of the occlusal face, and (C) final radiography to verify the quality of the endodontic and restorative treatment.

Finally, a base in glass ionomer cement was inserted before the definitive restoration in CR of the occlusal surface and final radiography (Figure 4) to verify the quality of the endodontic and restorative treatment.

Clinical-radiographic observations were performed after the third session using photographic documentation (Canon 60D, Canon do Brasil, São Paulo, SP) and periapical radiographs. The observation periods were seven days, three months, six months, nine months, and five years.

During the seven-day, three-month, six-month, and nine-month follow-ups, clinically good periodontal health, preservation of space for the supracrestal insertion tissue, absence of bleeding on probing, gingival edema, absence of marginal infiltration, absence of premature contact, adequate adaptation and integrity of the restoration, and absence of fractures or cracks in the tooth and/or restoration were observed.

**Figure 5:**
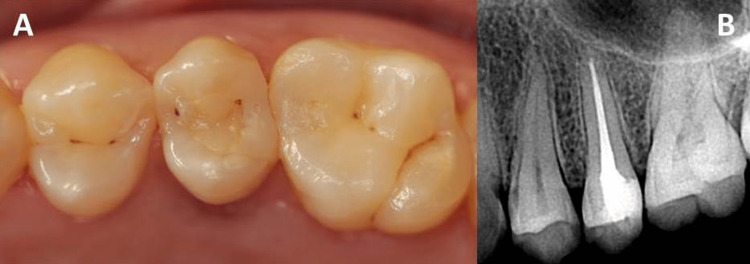
Follow-up five years: (a) the restoration was in good adaptation and conservation, (b) the successful endodontic treatment and the healing of the periradicular tissues.

The 12-month follow-up, which would allow for a more secure and proper evaluation of the regression of the periradicular lesion, was not performed as the patient did not respond to calls. This was only possible five years after the completion of the procedure. At that time, the asymptomatic dental element was clinically observed to be in perfect function, the restoration was in good adaptation and conservation, and radiographically, the successful endodontic treatment was present and confirmed (Figure 5), given the absence of a periapical lesion and the healing of the periradicular tissues.

## Discussion

Adequate rehabilitation of the dental element after completion of endodontic therapy has always been a concern in the literature, having been referred to as one of the most important aspects in endodontic success [14].

Clinical trials, systematic reviews, and meta-analyses that have evaluated the reverse sequence, in which RT is performed prior to ET, were not found in the literature.

The reversal of the order of procedures performed in this case report is not a new technique but a proposal for alteration as a way of reconstructing lost dental tissue, minimizing the rates of tooth loss caused by a fracture in teeth to be endodontically treated [15]. If teeth had their reinforcing structures reconstructed before endodontic treatment, the probability of the tooth remaining in the mouth would increase significantly, even if there was a delay in the performance of the definitive coronal restoration [15]. This would also reduce the need for endodontic retreatment, as a temporary or inadequately sealed coronal restoration can cause tooth reinfection [16] due to the lack of adhesion of the sealing material and compatibility with the dental structure.

In the present case, there was a need for surgery to restore the supracrestal insertion space. Restorations placed below the gingival margin are harmful to gingival and periodontal health [17]. In a longitudinal study with follow-up for a period of 26 years, the authors suggest that the increase in clinical attachment loss found in teeth with subgingival restorations began slowly and could be clinically detected one to three years after the execution of the restorations [13].

For ET, a sequence of treatment planning in endodontics was established based on the diagnosis of chronic apical periodontitis, which determines the indication of ET. This treatment could be performed in just one clinical session. However, the decision to perform prior restorative treatment, with the need for recovery of the biological width, made the consultation time very long and tiring for the patient. Thus, in the first session, only the coronal access and catheterization of the root canals were performed, with the application of an intracanal medication (tricresol formalin in a cotton pellet applied to the pulp chamber) with appropriate volatility action for situations like this, where root canal instrumentation was not performed [18].

In the third session, the ET was properly completed, performing instrumentation and obturation of the root canals. The pre-endodontic restoration performed in the first session allowed for the absolute isolation to be performed quite easily and safely, with the correct adaptation of the clamp and rubber dam in the cervical region of the teeth, leaving the area free of contaminating oral fluids and preventing contact of the oral mucosa with hypochlorite, without the need for additional techniques to ensure proper isolation.

Reinforcing structures, such as marginal ridges and cusps, are primarily responsible for the resistance of teeth, and their reconstruction is extremely important for maintaining the integrity of the remaining teeth [19]. In addition, pre-endodontic restorations can also help reduce the occurrence of dental fractures. A weakened tooth is more susceptible to fractures under pressure, which can be aggravated by the stress of root canal therapy. By placing a pre-endodontic restoration, the tooth is strengthened, reducing the risk of fracture during and after canal treatment [9].

The decision to restore the tooth using only a direct composite restoration is justified by the fact that it is a simplified and feasible procedure to recover the biomechanical properties of endodontically treated premolars with the structural loss [20], based on the principles of minimally invasive dentistry [21]. The use of a fiber post could have been a clinical alternative, however, it was clinically demonstrated that post-placement has no influence on reducing the failure of post-endodontic restorations and might not be necessary in teeth with some structure preserved [22]. Furthermore, adhesion to intraradicular dentin is less predictable than adhesion to coronal dentin [23], and the currently available luting agents cannot hermetically seal the endodontic cavity, which may affect the longevity of the adhesive interface and reduce the durability of the restoration [24].

Teeth with conservatively endodontic cavities that preserve the integrity of the marginal ridge have greater fracture resistance, and upper premolars restored with composite resin exhibit better force distribution [10]. These findings can be compared to another study where teeth with mesio-occlusal-distal (MOD) cavities became severely weakened due to the loss of reinforcing structures, such as marginal ridges and pulp chamber roofs, facilitating the fracture process when there was a delay in performing the definitive coronal restoration [25]. This highlights the importance of restoring lost dental structure through CR restoration before even performing the endodontic treatment, as it provides reinforcement of the remaining tooth structure.

In clinical situations where extensive MOD cavity preparations occur in premolars, the fracture resistance of the remaining cusps is reduced to one-third when compared to healthy teeth [26]. In another study, teeth that contained structural loss on the occlusal and palatal surfaces had their fracture resistance reduced by around 60% when compared to healthy teeth. These findings corroborate with the results of the study that evaluated the resistance of fifty-five recently extracted, intact, unrestored upper premolars subjected to structural losses, where the group of teeth with structural loss only on the occlusal surface had the highest fracture resistance [1]. This shows that endodontic access with structural loss that compromises the tooth circumference substantially weakens the remaining tooth structure [27].

Regarding the surfaces most prone to fracture, the palatal cusps of upper premolars tend to fracture more frequently than the vestibular cusps [28]. This is likely due to them being the working cusps, therefore subject to a greater chewing load.

Endodontically treated teeth are more prone to clinical failures when proximal contacts are not properly re-established with adjacent teeth [27]. This finding was confirmed in the literature, where potentially associated factors for the failure of endodontically treated teeth were identified. Among them, the absence of proximal contacts (26.2%) compared to teeth that had one (13.3%) or two (11.5%) proximal contacts preoperatively. This is caused by inefficient provisional sealing due to the lack of structure for proper retention of the provisional material, making the tooth more susceptible to recontamination, a situation aggravated by the delay in performing the definitive restoration [16].

It has been observed that the main reasons for the extraction of endodontically treated teeth were periodontal disease, endodontic failure, and non-restorable tooth damage caused by fractures or caries [29]. Therefore, the selection of an ideal restorative modality to compensate for the loss of coronal tooth structure is considered the key to the success of post-endodontic restorations [30].

Restorations performed with CR have the advantage of being adhesive to the tooth structure, which can strengthen the tooth, thus offering an alternative technique to indirect restoration. A study with upper premolars found that, after restoration with composite resin, these teeth largely recovered their fracture resistance, being only less resistant when compared to intact teeth. Thus, the choice of restorative material used in the present study was adequate, since the tooth presented integrity in the restoration performed and good marginal adaptation during all follow-up evaluations, including five years [31].

For a restoration with composite resin to have a good outcome, a strong and durable bond between the composite and dentin through the adhesive is necessary. NaOCl has no effect on dentin's inorganic part. However, it has a significant effect on the organic component of dentin [32]. The use of NaOCl during the biomechanical preparation of the root canals alters the surface structure of dentin and partially removes the collagen fibrils, interfering with the interaction with adhesive restorative materials, compromising thus, the formation of a consistent hybrid layer [33] and consequently, the adhesion of dentin with the composite resin [11], being this another disadvantage in performing RT only after ET.

The NaOCl interferes by reducing the bond strength between composites and dentin [34]. The presence of residual oxygen in dentin can affect the polymerization of adhesives, thereby making the bond interface less resistant and more vulnerable to degradation. It has also been found that the bond strength of adhesives (Single Bond and Scotchbond Universal, 3M, Sumaré, Brazil) to NaOCl-treated dentin was significantly lower than that of the respective control group (distilled water). This compromised bond is due to the fact that NaOCl is an oxidizing substance, leading to strong inhibition of interfacial polymerization of adhesive materials [11,35,36]. Another possible cause for the reduction in bond strength has been attributed to the presence of residual NaOCl in dentinal tubules interfering with the penetration of resin adhesive into the dentin or the polymerization of resin monomer [33]. These are other scientific evidences for performing restorative procedures in composite resin prior to the use of NaOCl in endodontic treatments. The use of glass ionomer cement as a base for sealing the coronal endodontic access, as performed in this clinical case, could be a good clinical alternative to the deleterious effects of hypochlorite on dentin and the adhesive interface, due to its chemical adhesion to the inorganic, unaltered portion of dentin [37].

Currently, it is recommended that the newly-obturated root canal be sealed quickly and effectively by making a definitive restoration to maintain periapical health since the presence of a temporary or inadequately sealed coronal restoration can lead to reinfection of the tooth [17]. Sometimes, the failure to perform definitive restorations immediately after the completion of root canal treatment can compromise the success of the treatment and the maintenance of the dental element in function [15].

Given this need for immediate restoration after root canal treatment, a study conducted at a Public Reference Center for Endodontics (Regional CEO, Sobral, Ceará, Brazil) found that 32.4% of patients undergoing treatment were not called by the public unit that referred them for definitive post-endodontic restoration, even after a minimum period of six months. This result demonstrates that the reference and counter-reference model based on the public health guidelines of this country does not favor the resolution of treatments of this nature. Teeth remain with temporary restorations and without the restoration of the coronal structure, which ends up favoring dental fracture. When not referred for extraction, they require new endodontic intervention [15]. This finding confirms the need to change the paradigm of treatment sequence, in which the restorative approach is performed before and after root canal treatment, as presented in this work.

It is a fact that the restorations performed after the completion of endodontic treatment are important in the success of the treatment [19]. The combination of root canal treatment with proper coronal restoration favors the success of treatment, regardless of the quality of the root canal treatment. The combination of proper coronal restoration with inadequate root canal treatment presented success rates of 83.33%. However, the combination of inadequate coronal restoration and adequate root canal treatment presented a percentage of 58.33% [15]. Thus, clinical failures are generally not related to the consequences of root canal treatment but to inadequate restorative therapy or periodontal reasons [38].

The reported case aimed to add the question of the need to include pre-endodontic restorations in this "equation." Such a procedure has been shown to be crucial for maintaining the longevity of teeth and reducing the risk of dental fractures, as they provide a solid foundation for successful root canal therapy and help ensure that the natural tooth can be preserved for as long as possible [9].

Clinical trial studies, systematic reviews, and meta-analyses that investigate this variable within the suggested context should be conducted to assess the level of importance of this procedure in maintaining dental elements, reducing losses due to fractures, and clinical failures. It is hoped that this will confirm the positive influence of pre-endodontic restorations in reducing dental fractures and improving endodontic success.

## Conclusions

Based on the observed case result after five years, it can be concluded that the tooth presented with periodontal health, the composite resin restoration was in good adaptation and conservation, and the periradicular region was healed.

Therefore, the pre-endodontic restorative treatment was resilient, contributing to the maintenance of the tooth in function without dental fracture and to the success of endodontic therapy. Clinical trials, systematic reviews, and meta-analyses should be conducted to increase the level of scientific evidence related to this issue.

## Figures and Tables

**Figure 6:**
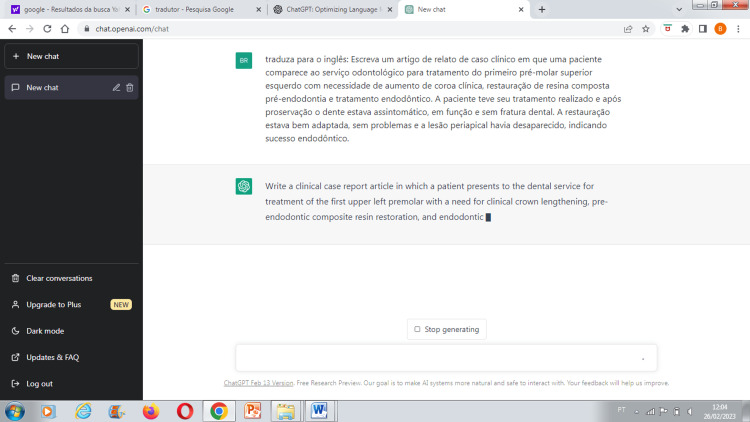
Article by ChatGPT.

**Figure 7:**
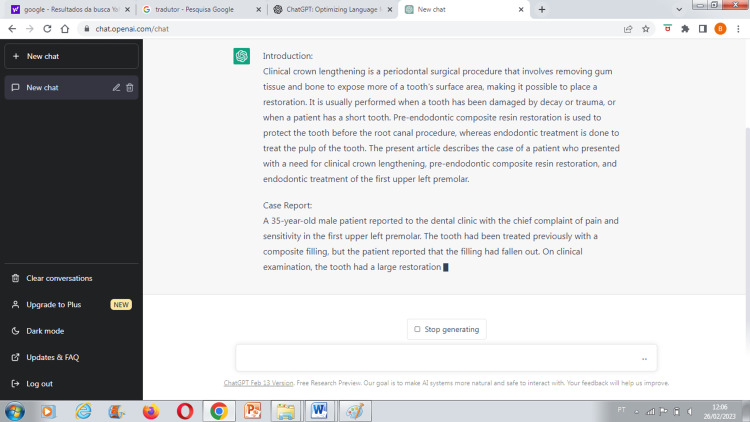
Print introduction by ChatGPT.

**Figure 8:**
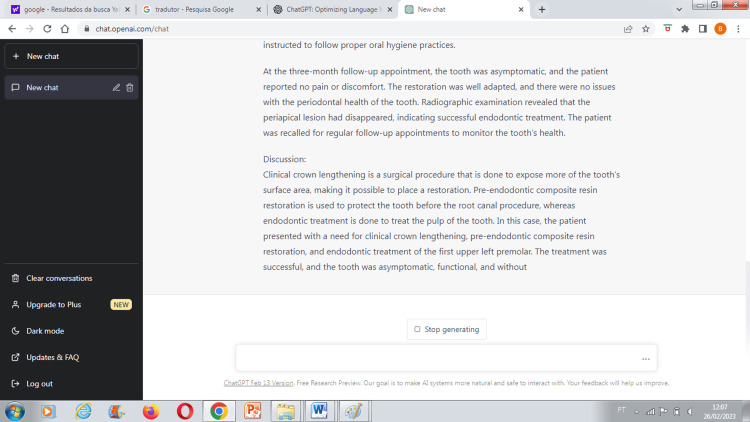
Print discussion by ChatGPT.

**Figure 9:**
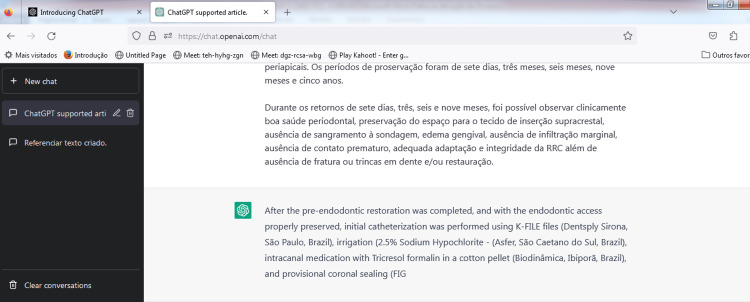
Print translate by ChatGPT.

## Appendices

Use of ChatGPT artificial intelligence (https://www.chatgpt.com) to assist in the article's development, as a research tool and search for arguments used in the article's introduction and discussion, and in translating the article into the English language (Figures 6, 7, 8, 9).
